# Impact of combining intermittent preventive treatment with home management of malaria in children less than 10 years in a rural area of Senegal: a cluster randomized trial

**DOI:** 10.1186/1475-2875-10-358

**Published:** 2011-12-13

**Authors:** Roger CK Tine, Babacar Faye, Cheikh T Ndour, Jean L Ndiaye, Magatte Ndiaye, Charlemagne Bassene, Pascal Magnussen, Ib C Bygbjerg, Khadim Sylla, Jacques D Ndour, Oumar Gaye

**Affiliations:** 1 Service de Parasitologie, Faculté de Médecine et Pharmacie, Dakar, Senegal; 2 Clinique des Maladies Infectieuses, Centre Hospitalier Universitaire de Fann, Dakar, Senegal; 3 DBL - Centre for Health Research and Development, Faculty of Life Sciences, University of Copenhagen, Copenhagen, Denmark; 4 Department of International Health, Immunology and Microbiology, Faculty of Health Sciences, University of Copenhagen, Copenhagen, Denmark; 5 Ministére de la santé et de la prévention du Sénégal, District sanitaire de Velingara, Velingara, Dakar, Senegal

**Keywords:** Malaria, Intermittent preventive treatment, Home-based management, Anaemia

## Abstract

**Background:**

Current malaria control strategies recommend (i) early case detection using rapid diagnostic tests (RDT) and treatment with artemisinin combination therapy (ACT), (ii) pre-referral rectal artesunate, (iii) intermittent preventive treatment and (iv) impregnated bed nets. However, these individual malaria control interventions provide only partial protection in most epidemiological situations. Therefore, there is a need to investigate the potential benefits of integrating several malaria interventions to reduce malaria prevalence and morbidity.

**Methods:**

A randomized controlled trial was carried out to assess the impact of combining seasonal intermittent preventive treatment in children (IPTc) with home-based management of malaria (HMM) by community health workers (CHWs) in Senegal. Eight CHWs in eight villages covered by the Bonconto health post, (South Eastern part of Senegal) were trained to diagnose malaria using RDT, provide prompt treatment with artemether-lumefantrine for uncomplicated malaria cases and pre-referral rectal artesunate for complicated malaria occurring in children under 10 years. Four CHWs were randomized to also administer monthly IPTc as single dose of sulphadoxine-pyrimethamine (SP) plus three doses of amodiaquine (AQ) in the malaria transmission season, October and November 2010. Primary end point was incidence of single episode of malaria attacks over 8 weeks of follow up. Secondary end points included prevalence of malaria parasitaemia, and prevalence of anaemia at the end of the transmission season. Primary analysis was by intention to treat. The study protocol was approved by the Senegalese National Ethical Committee (approval 0027/MSP/DS/CNRS, 18/03/2010).

**Results:**

A total of 1,000 children were enrolled. The incidence of malaria episodes was 7.1/100 child months at risk [95% CI (3.7-13.7)] in communities with IPTc + HMM compared to 35.6/100 child months at risk [95% CI (26.7-47.4)] in communities with only HMM (aOR = 0.20; 95% CI 0.09-0.41; *p *= 0.04). At the end of the transmission season, malaria parasitaemia prevalence was lower in communities with IPTc + HMM (2.05% versus 4.6% *p *= 0.03). Adjusted for age groups, sex, *Plasmodium falciparum *carriage and prevalence of malnutrition, IPTc + HMM showed a significant protective effect against anaemia (aOR = 0.59; 95% CI 0.42-0.82; *p *= 0.02).

**Conclusion:**

Combining IPTc and HMM can provide significant additional benefit in preventing clinical episodes of malaria as well as anaemia among children in Senegal.

## Background

Malaria remains a major public health problem in tropical regions. According to the World Health Organization (WHO), in 2009 there were an estimated 169-294 million cases and 628,000-968,000 deaths worldwide. Over 89% of these deaths occur in Africa [1], most of the time outside health facilities [2,3]. In view of this situation, there is need to strengthen malaria case management and malaria prevention at the community level to reduce the burden of disease. Recently, the WHO advocated for scaling up malaria control interventions in order to accelerate malaria elimination [4] Several malaria control strategies were developed recently, including (i) early case detection using rapid diagnostic tests (RDT), prompt treatment with effective anti-malarial drugs, such as artemisinin combination therapy (ACT) for uncomplicated malaria cases, (ii) pre-referral rectal artesunate for severe malaria cases, (iii) intermittent preventive treatment, and (iv) long-lasting insecticide-treated nets (LLIN).

Effective case management is a fundamental element of malaria control. To improve treatment practices at community level, the strategy of home-based management of malaria (HMM) has been developed [5,6]. Home-based management of malaria (HMM) is now considered as an important strategy for reducing severe morbidity and mortality from malaria in resource-poor countries [7,8].

In Senegal, the National Malaria Control Programme (NMCP) has initiated the scaling up of the use of ACT at community level, in the context of HMM strategy, implemented by community health workers (CHWs) in order to strengthen malaria control efforts. This strategy includes the use of RDT for malaria confirmation and ACT for the treatment of uncomplicated malaria.

Intermittent preventive treatment (IPT) is a new approach aiming at reducing malaria morbidity among children or other high-risk individuals. IPT involves administration of anti-malarial drugs at defined time intervals to individuals regardless of whether they are known to be infected with malaria to prevent morbidity and mortality from the infection [9]. IPT was initially recommended for pregnant women involving the administration of at least two doses of sulphadoxine-pyrimethamine (SP) during antenatal visits after the first trimester of pregnancy. More recently the strategy was extended to infants (IPTi) with the administration of three doses of an anti-malarial drug during the expanded programme of immunization (EPI) visits [10]. In children under 5 years of age, several studies have shown IPT to be effective in reducing malaria burden [11,12]. Intermittent preventive treatment of malaria in children less than 5 years of age (IPTc), involves the administration of two to three doses of anti-malarial drug during the high malaria transmission season [13].

Cissé *et al*. during a randomized double-blind controlled trial, conducted in Niakhar in Senegal have shown that administering SP plus artesunate three times during the transmission season can reduce malaria incidence among children under 5 years by 86% [11]. Protective efficacy of IPTc in Mali was estimated at 67.5% with two doses of SP at 8 weeks intervals during the high malaria transmission season [12]. Another study conducted in the rural area of Niakhar (Senegal) demonstrated that the most optimal regimen for IPTc in children is the combination of SP-amodiaquine. To ensure a maximum protective effect, the IPTc should preferably combine two long half-life drugs [14].

In most African countries, anti-malarial interventions are being promoted on an individual basis and many communities are still not getting access to those services [15]. In 2008, it was estimated by WHO that confirmation of malaria cases was done in only 22% on average in most African regions, while less than 15% of patients under 5 years of age suffering from malaria attacks benefitted from treatment with ACT. It thus appears that the use of effective malaria control strategies and their integration into national health systems and services continue to be a challenge in Africa [1]. In addition, individual anti-malarial interventions provide only partial protection in most epidemiological situations [16,17]. Therefore, there is a need to investigate the potential benefits of integrating several malaria interventions, in reducing malaria prevalence and morbidity. This study aimed to assess the impact of combining seasonal intermittent preventive treatment in children (IPTc) with home-based management of malaria (HMM) by community health workers (CHWs) in Senegal.

## Methods

### Study area and population

The study was carried out at the Bonconto health post, located at the Velingara health district in the south-eastern part of Senegal, 500 km from the capital city of Dakar. The health post is headed by a nurse and has eight functional health huts staffed with community health workers, serving a total population of 10,016 inhabitants. In this area malaria transmission is seasonal, occurring during the rainy season (July to November) with a peak transmission in October and November. *Plasmodium falciparum *is the predominant parasite species and transmission is mainly due to *Anopheles gambiae s.l*. (Konate Lassana, personal communication). In this area, the National Malaria Control Programme initiated the universal coverage of LLIN strategy in 2010.

### Study design

The study was designed as a cluster randomized trial. Eight CHWs in the eight villages around the Bonconto health post, were trained to diagnose malaria using RDT and provide prompt treatment with artemether-lumefantrine to children less than 10 years. Four of them were randomized to also administer monthly IPTc with single dose of SP plus three doses of amodiaquine (AQ) in October and November 2010. The randomization unit was the CHW in order to avoid contamination. Each CHW is covering one village. The CHWs were randomized using a random number generator from Excel software.

Primary end point was incidence of single or first malaria attack over 8 weeks of follow up throughout an active surveillance system. Malaria attack was defined as presence of fever (temperature > 37.5°C) with a positive RDT. Secondary end points were prevalence of malaria at the end of the transmission season, and prevalence of anaemia at the end of the transmission season in the two groups.

### Interventions

The two main interventions in this study were HMM and IPTc for children aged from one to 10 years. During the study period, RDT were deployed at the level of health huts. The RDT used in this study was based on the detection of the Histidine Rich Protein II (Malaria Antigen P.f SD^®^), and was provided by the NMCP.

For uncomplicated malaria cases, treatment was done by CHWs using artemether-lumefantrine according to age group; children presenting severe malaria cases received one dose (10 mg/kg) administration of pre-referral artesunate suppositories prior to their transfer to the Bonconto health post. In the four villages with combined HMM and IPTc, all doses of AQ and SP were administered by CHWs under direct observation. IPTc drug delivery was organized at the level of health huts. At scheduled days for IPTc administration, parents were asked to bring their child at the health huts for IPTc delivery. In case a child was not seen at time of administration, the CHW was advised to visit that child at home and give the treatment. To facilitate SP and AQ administration, treatment doses were tabulated on a document and distributed to each CHW to serve as job aid.

Each tablet of AQ contains 153 mg of amodiaquine base while SP tablet contains 500 mg sulphadoxine and 25 mg pyrimethamine. Treatment doses were according to age group. Children under 2 years of age received half a tablet of SP; a whole tablet of SP was given to children age from two to 6 years, while children age from seven to 10 years received one tablet and half of SP. For AQ half a tablet was given to children under 2 years, one tablet and one and a half tablet were given daily for 3 days to children aged 2-7 years and 8-10 years respectively. This drug regimen has been shown to be the most optimal regimen to minimize overdosing as well as under dosing of AQ [18].

Artemether-lumefantrine (Novartis^LTD^) was provided by the Senegalese NMCP, rectal artesunate was obtained from Mepha^Ltd^, while AQ and SP were provided by Kina Pharm^Ltd^.

### Data collection

#### Baseline assessment

Prior to start of the study, meetings were held in the villages to explain the study purpose and answer to the population's questions. Consent was obtained from the community leaders as well as parents or children's guardians. A census of all children aged from one to 10 years leaving in each randomized village was done. A baseline assessment was done prior to the intervention (beginning of October). At baseline, all registered children were examined by a study physician, and their mothers interviewed to assess the use of bed nets, and the presence of any chronic illness which might interfere with the outcome of the trial. Thick and thin blood films were prepared and haemoglobin concentration measured using HemoCue Hb 201^®^. Children with acute malaria during the baseline study (temperature > 37.5°C and positive RDT) were treated with artemether-lumefantrine and those with anaemia (Hb < 11 g/dl) received oral iron supplementation for 1 month.

#### Malaria cases detection

An active surveillance system was organized in the eight villages from the date of first IPTc administration, to the end of the transmission season in December. Children were visited at home by CHW once a week during 8 weeks. At each visit children's axillary temperature was measured. If the child had fever (temperature > 37.5°C), or a history of fever within the previous 24 h, a RDT was performed by the CHW. Children with acute malaria (temperature > 37.5°C and positive RDT) received a three-day treatment with artemether-lumefantrine. A follow-up was done by the CHWs up to day seven after treatment, to monitor the patient's clinical conditions. In case the child did not recover on day 3, CHWs were advised to refer the child to the health post. The mothers were encouraged to take their child to the CHWs if the child presented fever in non-scheduled days visits.

#### Malaria parasitaemia and anaemia prevalence evaluation

A cross-sectional survey was carried out at the end of the malaria transmission season in a subsample of study participants, randomly selected from the list of children less than 10 years living in the eight villages. For each randomly selected child, thick and thin smear test were done, haemoglobin (Hb) concentration measured and anthropometric data collected.

#### Sample size calculation

With four clusters in each intervention arm and 125 children less than 10 years sampled in each cluster, assuming a current incidence of malaria attacks of 35 per 100 person-month at risk, the study was powered at 80% to detect 20% of reduction of malaria incidence in the HMM + IPTc group at 5% significance level, with a coefficient of variation of 0.3. For the cross sectional survey the total number of children to examine was calculated at 800, based on a prevalence of malaria parasitaemia at 20% in the study area (Senegal MIS 2009) a confidence level at 95% with a precision of 5%, power level at 90% and assuming a percentage of 20% of withdrawal.

### Laboratory methods

Blood samples were collected using finger prick blood. The first drop was used for thick and thin smear test for the diagnosis of malaria. Thick and thin smear test were stained with Giemsa and read by a laboratory technician. Malaria parasitaemia was defined as any asexual parasitaemia detected on a thick or thin blood smear. Parasite density was determined by counting the number of asexual parasites per 200 white blood cells, and calculated per μL using the following formula: numbered parasites × 8,000/200 assuming a white blood cell count of 8,000 cells per μL. Absence of malaria parasite in 200 high power ocular fields of the thick film was considered as negative.

The second drop of finger prick blood was drawn into a microcuvette for Hb determination (g/dl) using HemoCue machine (HemoCue^® ^Hb 201). Moderate and severe anaemia were defined as Hb concentration below 11 g/dl and 8 g/dl, respectively.

### Data analysis and data management

Data were entered in Excel™ software and analysed using STATA 11™ software. For descriptive data, percentage was used to assess the frequency of each outcome. For quantitative data, mean and standard deviation were used to describe normally distributed variables, median and range for other data. Characteristics of all children included in the study were tabulated by study arm.

For the primary end point of incidence of malaria attacks over 8 weeks of follow up, analysis was by intention to treat including all children who attended the baseline survey. For the secondary end points of cross sectional prevalence of malaria and anaemia at the end of the transmission season, analysis was done by per protocol, including all children seen at cross sectional survey at the end of the transmission season.

To assess the impact of combining HMM + IPTc, analysis was conducted at the individual level with adjustment for clustering using robust standard errors [19]. Time at risk was calculated from date of the first IPTc administration to the date of the cross-sectional survey. Children were not considered at risk for 28 days after treatment for malaria attacks, and thus were censored from the analysis for 4 weeks, although no child presented more than one malaria episode. Time at risk to the first malaria episode between the two study arms was compared using Kaplan Meier method with a log rank test stratified by clusters. The incidence rate ratio (IRR) of HMM + IPTC and HMM alone was determined after adjustment by age group and gender using Cox regression model with robust standard errors to account for clustering. The protective efficacy of HMM + IPTc on malaria incidence was calculated as (1-IRR) × 100. *P *values below 5% were considered as significant (two sided). Prevalence of malaria parasitaemia and anaemia at the end of the transmission season were measured in the two groups and compared using a logistic regression analysis with robust standard errors to take into account for the cluster design.

### Ethical considerations

Prior to the study, a community sensitization was undertaken and community consent was obtained from community leaders (religious guide, village head). Informed consent was obtained from parents or children's guardians the days of surveys. The study protocol was approved by the Senegalese National Ethical Committee (Conseil National de Recherche en Santé). Approval N 027/MSP/DS/CNRS, 18/03/2010.

## Results

### Baseline characteristics

**Figure 1:**
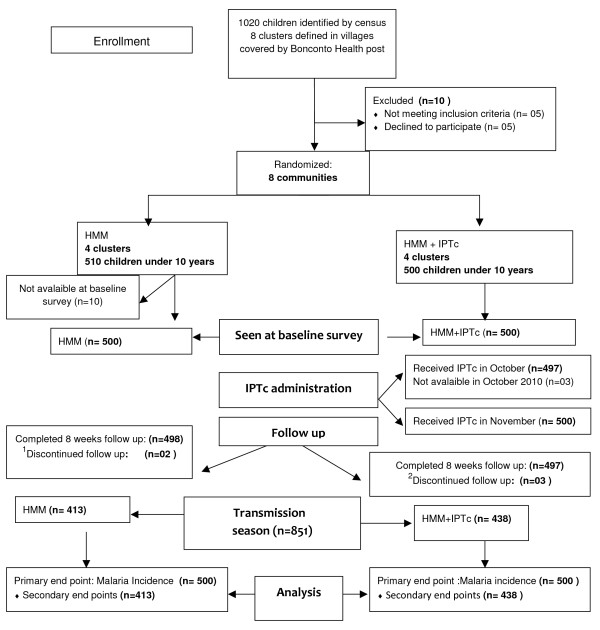
**Trial profile**: ^1^Two children in the HMM group were not seen by the CHWs during the second week of home visit. ^2^One child in the HMM + IPTc group was not seen during the first week of follow up and two during the second week.

One thousand and twenty children aged from 1 to 10 years were registered in the eight villages with functional health huts, covered by the Bonconto health post; 1,000 children (500 in the HMM group and 500 in the HMM + IPTc group) who met the entry criteria were enrolled (Figure 1).

At baseline the two groups were similar in terms of demographic characteristics (age, gender and *P. falciparum *carriage). Prevalence of moderate anaemia and severe anaemia were similar in the two groups, as well as prevalence of under nutrition (stunting, underweight); 95.8% of study subjects in the HMM + IPTc group and 95.4% in the HMM group slept under a LLIN (Table 1).

**Table 1:** Baseline characteristics of children in the two groups

Interventions		
**Parameters**	**HMM+IPTc**	**HMM**

Number of children	500	500

Number of clusters	04	04

Mean cluster size (range)	133 (130-145)	120 (102-145)

Age group		

Children under 5 years, %	57.4 (287/500)	50 (250/500)

Children aged from 5 to 10 years, %	42.6 (213/500)	50 (250/500)

Gender, % of male	45.2 (226/500)	51.6(258/500)

*P. falciparum *carriage, %	11.2 (56/500)	9.4 (47/500)

Moderate anaemia, % (Hb between 11 and 8 g/dl)	58 (290/500)	56.4(282/500)

Severe anaemia (Hb * < 8 g/dl), %	21 (105/500)	24.8(124/500)

Stunting (HAZ < -2SD**), %	46.4 (232/500)	41.2 (206/500)

Bed net usage, %	95.8 (479/500)	95.4 (477/500)

*Hb haemoglobin; *HAZ *height for age z score

### Impact of the interventions on malaria incidence

**Figure 2:**
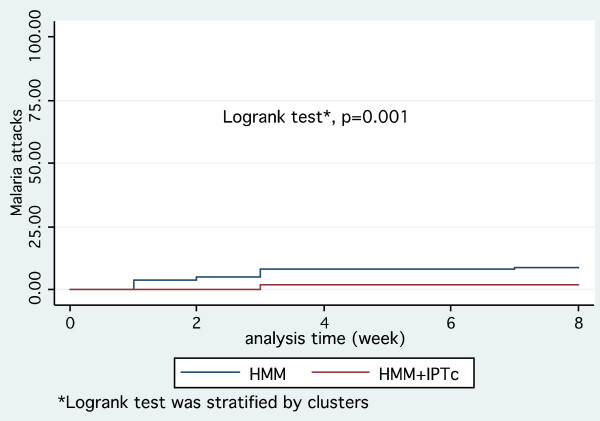
**Kaplan-Meier plot comparing time to first episode of malaria attack defined as fever (> 37.5°C) and positive RDT, between the two groups**.

Overall, the cumulative incidence of malaria episodes was significantly lower in the HMM + IPTc group. Thus, the Kaplan Meier survival estimates of time to first malaria episode showed a significant difference between the two groups (*p *= 0.001, log rank test) (Figure 2).

The incidence of clinical malaria attacks during the study period was 35.6 per 100 children-months at risk in the HMM group while that for children in the HMM + IPTc group was only 7.2 per 100 children-month at risk (*p *= 0.04). After controlling for age group, and gender, the combination of IPTc + HMM significantly reduced the number of malaria episodes in children: adjusted incidence rate ratio: 0.21 (95% CI [0.10-0.42]); *p *= 0.04. Thus, the protective efficacy of IPTc + HMM against malaria attacks incidence (all cases) was 79% (95% CI [58%-90%]) (Table 2). During the intervention period, 5/510 children in the HMM group (0.98%) presented severe malaria, while no severe malaria cases were noted in the HMM + IPTc group.

**Table 2:** Impact of IPTc combined to HMM on malaria incidence in the two groups

	Malaria incidence Rate/100 person-month (95% CI)	Unadjusted IRR (95% CI)	Adjusted IRR (95% CI)	Protective efficacy (%) (95% CI)	p value*
**Interventions**					

**HMM**	35.5 [25.0-50.3]	1	1		

**HMM + IPTc**	07.1 [1.1- 9.5]	0.20 [0.04-1.0]	0.21[0.04-0.90]	79 [10; 96]	0.04

**Gender**					

**Female**	21.0 [11.1-44.3]	1	1		

**Male**	22.4 [11.6-48.3]	1.06 [0.62-1.84]	1.18 [0.74-1.86]	-18[-86;26]	0.48

**Age groups**					

**1 years**	17.3 [10.1-32.8]	1	1		

**[2 - 5 years]**	28.5 [13.8-66.3]	1.62 [0.84-3.10]	1.50[0.85-2.65]	-50[-165;15]	0.16

**[6- 10 years]**	17.4 [09.8-32.8]	1.0[0.74-1.34]	0.98[0.74-1.27]	2 [-27; 26]	0.85

*HMM *home-based management; *HMM+IPTc *home-based management+intermitent preventive treatment; *IRR *incidence rate ratio. Intraclass correlation coefficients for Gender:0.01, age group:0.007. *All p value are adjusted for clustering by robust standard errors.

### Impact of the interventions on malaria parasitaemia at the end of the transmission season

At the end of the malaria transmission season, 28 children (3.3%) were found with *P. falciparum*. A proportion of 4.6% (95% CI [2.5-6.6]) children in the HMM group had asexual *P. falciparum *(any density) compared with 2.1% (95% CI [0.7-3.3]) in the HMM + IPTc group. The proportion of children with *P. falciparum *parasitaemia at any density, was significantly lower in the HMM + IPTc group (OR = 0.43 (95% CI [0.19-0.95]); *p *= 0.03), thus IPTc + HMM had a protective efficacy against *P. falciparum *parasitaemia (at any density) of 57% (95% CI [5%-81%]).

Children with parasitaemia at a density > 1,000 parasites/μL at the end of the transmission season, represented 3.39% and 1.37% in the HMM and HMM + IPTc groups, respectively (OR = 0.40 (95% CI [0.16-0.96]); *p *= 0.05) resulting in a protective efficacy at 60% (95% CI [04%-84%]) (Table 3).

**Table 3:** Impact of interventions on malaria parasitaemia at the end of the transmission season

	HMM (n = 413)	HMM + IPTc (438)	OR (95% CI)	Protective efficacy (%)	p value
***P. falciparum *parasitaemia**					

**> 1,000 parasite/μL**	14 (3.39%)	6 (1.37%)	0.40 [0.16 - 0.96]	60% [04 - 84]	0.05

**any density**	19 (4.60%)	9 (2.05%)	0.43[0.19-0.95]	57% [05 - 81]	0.03

### Impact of the interventions on anaemia prevalence at the end of the transmission season

Mean Hb concentration among children less than 10 years of age at the end of the malaria transmission season was 10.4 ± 1.98 g/dl in the HMM + IPTc group and 10.2 ± 1.8 g/dl in the HMM group (*p *= 0.07). Proportion of anaemic children (hb < 11 g/dl) at the end of the transmission season was 54.11% in HMM + IPTc group, while anaemic children represented 60.3% in the HMM group (*p *= 0.06). In a logistic regression analysis with robust standard errors, HMM + IPTc showed a significant protective effect against anaemia (adjusted Odds Ratio (aOR): 0.59 (95% CI [0.42-0.82]); *p *= 0.025. The protective efficacy of HMM + IPTc in reducing anaemia among children under 10 years was estimated in this study at 41% (95% CI 95 [18%-58%]).

Anaemia was also significantly associated with *P. falciparum *carriage at the end of the transmission season, (aOR = 2.57; 95% CI [1.1-6.70]; *p *= 0.026), stunting (aOR = 2.97; 95% CI [2.08-4.23]; *p *= 0.001), age range from two to 5 years (aOR = 0.14; 95% CI [0.08-0.25]; *p *= 0.001) and age above 5 years (aOR = 0.04; 95% CI [0.02-0.07]; *p *= 0.001) (Table 4).

**Table 4:** Impact of interventions on anaemia prevalence at the end of the transmission season

Anaemia (Hb inf 11 g/dl)					
	**Participants (%)**	**OR (95% CI)**	**aOR (95% CI)**	**Protective efficacy (%) 95% CI**	**p value**

**Interventions**					

HMM (n = 411)	248 (60.3)	1	1		

HMM+IPTc (n = 438)	237 (54.1)	0.77[0.59 -1.02]	0.59[0.37-0.93]	41%[18;58]	0.025

**Gender**					

Female (n = 398)	213 (53.5)	1	1		

Male (451)	272(60.3)	1.32[1.01-1.73]	1.19[0.80-1.75]	-19%[-75;20]	0.35

***P. falciparum carriage***					

No (n = 821)	464 (56.5)	1	1		

Yes (n = 28)	21 (75.0)	2.30[0.83-6.37]	2.56[1.12-5.85]	-156%[-485;-12]	0.026

**Stunting (HAZ < -2SD)**					

No (n = 546)	265 (48.53)	1	1		

Yes (n = 305)	220 (72.13)	2.72 [1.86-3.98]	2.97[2.41-3.66]	-197[-323;-108]	0.001

**Age groups**					

Under 2 years n = 179)	162 (90.5)	1	1		

[2-5 years](n = 374)	233 (62.30)	0.17[0.09-0.31]	0.14[0.08-0.24]	86%[76;92]	0.001

[5-10 years] (n = 296)	88 (29.73)	0.04[0.02-0.08]	0.04[0.02-0.07]	96%[93;98]	0.001

Goodness of fit test: Hosmer Lemeshow, Chi 2 (8df) = 7,36, *p *= 0,49. *aOR adjusted odds ratio; *OR *odds ratio. Intra-class correlation coefficients for each outcome were as follows: age:0.02, Gender:0.001, stunting:0.08, slide microscopy positive:0.011. All p values are adjusted for clustering by robust standard errors.

## Discussion

Malaria remains a major public health problem in Africa, despite the decline in malaria incidence reported by most African countries in recent years [20]. Early case detection and prompt effective treatment with ACT are essential tools for malaria control. Intermittent Preventive Treatment (IPT) is a new approach aiming at reducing malaria morbidity and mortality. IPT is recommended by the WHO for pregnant women and infants. In children, the strategy is still debated and several studies are in progress [7-9].

This study assessed the potential benefit of combining home based management of malaria with IPTc in an area with high coverage of ITNs. The trial, conducted in a rural area in Senegal, where malaria is highly seasonal, showed that combination of IPTc and HMM can provide substantial benefit in reducing malaria. Indeed, malaria incidence was lower in villages where HMM was combined with IPTc compared to villages with only HMM strategy. *P. falciparum *carriage at the end of the transmission season was significantly lower in communities assigned to IPTc + HMM. No severe malaria cases were noted in the HMM + IPTc arm, while five severe malaria cases were registered in the HMM arm; thus the combination of IPTc and HMM can provide substantial benefit in reducing occurrence of severe malaria cases. The combined interventions also provided an additional benefit in reducing the occurrence of anaemia in children less than 10 years of age.

These results are consistent with data from other trials. Tagbor *et al*., in a randomized controlled trial conducted in children under 5 years in Ghana, demonstrated that combining IPTc with HMM can significantly reduce the incidence of malaria presumptive fevers [21]. Another trial in the Gambia [17] showed a reduction in the incidence of malaria in children under 5 years of age, when HMM is combined with IPT.

The expansion of malaria control measures at community level has been recommended by the WHO, in order to accelerate malaria elimination [4]. Malaria elimination will require the use of combination of interventions [17], and this study showed that community health workers can play an important role in scaling up anti-malarial interventions and even contribute to the malaria elimination process.

The study showed that combining HMM to IPTc in an area with high coverage of ITN (95%) will provide additional benefit in reducing malaria burden. The high coverage of ITN in the study area means that study participants had access to two or three interventions (HMM and ITNs or HMM, IPTc, ITNs). Thus, a third arm with only ITNs use would be appropriate to better understand the effect on malaria burden of several anti-malarial interventions. In other trials, conducted in Burkina Faso [22] and Mali, [23] IPTc showed a high level of protective efficacy against symptomatic malaria, severe malaria, as well as moderate and severe anaemia in children less than 5 years sleeping under ITNs. It thus appears that IPTc would provide a valuable contribution in reducing malaria by itself, or integrated with other intervention strategies, in areas with highly seasonal malaria [24].

The combination of IPTc and HMM was effective in reducing the magnitude of malaria and anemia in children less than 10 years. Although combined malaria control strategies at community level are likely to reduce malaria burden drastically, there are however, limited information on how the resultant drug pressure (IPTc drugs, ACT) may impact existing drug resistance. Consequently, it is important to monitor drug resistance while scaling up anti-malarial interventions at community level.

Although HMM + IPTc showed a significant protective effect against anaemia, the prevalence of anaemia at the end of the transmission season was still high. Anaemia was closely associated with *P. falciparum *carriage and stunting. It is thus important to implement community-based interventions to reduce anaemia among children in rural areas, to complement interventions against malaria, and malaria related anaemia. These interventions could include, among other things, strengthening and improving children's nutritional status and investigating for other possible causes of anaemia.

## Conclusion

Combining IPTc and HMM can provide significant additional benefit in preventing clinical episodes of malaria as well as anaemia among children in Senegal. IPTc would provide a valuable contribution in reducing malaria, by itself or integrated with other intervention strategies, in areas with highly seasonal malaria.

## Competing interests

The authors declare that they have no competing interests.

## Authors' contributions

RCT, CTN, PM, ICB, OG conceived and designed the study. RT, CB and KS trained CHWs, supervised the fieldwork and the data collection. RT analysed the data. RT, CTN, PM, ICB, OG, JLN, BF, MN, JDN wrote the manuscript. All authors read and approved the final manuscript.
